# Liver X receptor agonist treatment attenuates cardiac dysfunction in type 2 diabetic *db/db* mice

**DOI:** 10.1186/s12933-014-0149-0

**Published:** 2014-11-22

**Authors:** Qing He, Jun Pu, Ancai Yuan, Tianbao Yao, Xiaoying Ying, Yichao Zhao, Longwei Xu, Huan Tong, Ben He

**Affiliations:** Department of Cardiology, Ren Ji Hospital, School of Medicine, Shanghai Jiao Tong University, Shanghai, 200127 China

**Keywords:** Liver X receptor, Diabetic cardiomyopathy, Insulin resistance, Antioxidant

## Abstract

**Background:**

Liver X receptor (LXR) plays a critical regulatory role in metabolism and inflammation, and has been demonstrated to be involved in cardiovascular physiology/pathology. In the present study, we investigated the effect of GW3965, a potent LXR agonist, on diabetic cardiomyopathy (DCM) in type 2 diabetic *db/db* mice.

**Methods and Results:**

Non-diabetic *db/+* mice and diabetic *db/db* mice received either vehicle or LXR agonist GW3965 for 12 weeks. Systemic insulin resistance was evaluated by glucose tolerance test and homeostasis model assessment for insulin resistance. Endpoint cardiac function was assessed by echocardiography and catheterization. Ventricular tissue was collected for histology and gene/protein expression analysis. Untreated *db/db* diabetic mice exhibited diastolic dysfunction with adverse structural remodeling (including myocardial fibrosis and increased apoptosis). Treatment with GW3965 remarkably attenuated myocardial dysfunction and structural remodeling in diabetic *db/db* mice. Mechanistically, GW3965 restored Akt phosphorylation and inhibited MAP kinases phosphorylation, and reduced oxidative/nitrative stress and inflammation response in the diabetic myocardium.

**Conclusions:**

Our data demonstrate that GW3965 exerts a cardioprotective effect against DCM by (at least in part) attenuating insulin resistance, modulating Akt and MAP kinases pathways, and reducing oxidative/nitrative stress and inflammatory response. These findings strongly suggest that LXR agonist may have therapeutic potential in treating DCM.

## Background

The incidence and prevalence of diabetes mellitus are growing rapidly in societies around the world. Type 2 diabetes mellitus (T2DM) accounts for 90-95% of all diagnosed diabetes in adults [1]. Growing evidence has shown that diabetes mellitus, independent from other risk factors such as coronary artery disease and hypertension, can affect cardiac structure and function, which supports the existence of diabetic cardiomyopathy (DCM) [2]. As an independent diabetic cardiac complication, DCM is defined as diabetes-caused pathologic abnormalities including myocardial metabolic disturbance, oxidative/nitrative stress, inflammation, cardiomyocyte apoptosis, left ventricular dysfunction and structural remodeling [3]. Although treatment for DCM including improving glycemic control and restoring cardiovascular function is currently available to diabetic patients in clinical practice, therapeutic outcomes are far from satisfactory and the incidence of diabetes-induced cardiac dysfunction continues to escalate [3,4]. Thus, there is a great medical need to develop novel pharmacological or molecular interventions to treat left ventricular dysfunction and remodeling in DCM.

Liver X receptors (LXRs), including two different but highly homologous LXR isoforms (LXRα and LXRβ), are ligand-activated transcriptional factors belonging to the nuclear receptor superfamily [5]. LXRα is highly expressed in metabolically active tissues, such as liver, kidney, adipose, and intestines. LXRβ is ubiquitously expressed throughout the body [5]. Recently, by regulating metabolic and inflammatory pathways, LXR has been considered as a potential pharmacological target in the pathogenesis of cardiovascular and metabolic diseases [6]. Two synthetic LXR agonists, GW3965 and T0901317, have been reported to prevent atherosclerosis, inhibit inflammation, attenuate myocardial hypertrophy, and reduce ischemia/reperfusion injury [7-11]. Moreover, activation of LXR by T0901317 mitigates high glucose-induced oxidative stress, and apoptosis in cardiomyocytes *in vitro* [12]*.* However, the potential of LXR activation to attenuate the structural and functional defects caused by DCM *in vivo* have not been investigated. Therefore, the aims of the current study were to 1) investigate whether the LXR agonist GW3965 can protect the diabetic heart against adverse changes using the *db/db* mouse model of T2DM; and 2) clarify the downstream signaling mediating its effect in DCM.

## Materials and methods

### Reagents and antibodies

Synthetic LXR ligand 3-[3-[N-(2-Chloro-3-trifluoromethylbenzyl)-(2,2-diphenylethyl) amino] propyloxy] phenylacetic acid hydrochloride (GW3965) was kindly donated by Jon Collins (GlaxoSmithKline, Research Triangle Park, NC). Dihydroethidium (DHE) and TRIzol Reagent were from Life Technologies (Carlsbad, CA). Mouse monoclonal antibody against LXRα (ab41902) and rabbit polyclonal antibody against LXRβ (ab28479) were from Abcam (Cambridge, UK); Rabbit anti-mouse nitrotyrosine antibody (06-284) was from Millipore (Billerica, MA); rabbit anti-cleaved caspase-3 (5A1E, #9664), rabbit anti-nuclear factor kappa-light-chain-enhancer of activated B cell p65 (NF-κB p65, C22B4; #4764), rabbit anti-Akt (#9272), rabbit anti-phospho-Akt (D9E, Ser473, #4060), rabbit anti-p38 mitogen-activated protein kinase (p38 MAPK, #9212), rabbit anti-phospho-p38 MAPK (D3F9, Thr180/Tyr182, #4511), rabbit anti-c-Jun N-terminal kinase (JNK, 56G8, #9258), rabbit anti-phospho-JNK (81E11, Thr183/Tyr185, #4668), rabbit anti-Histone H3 (#9715) and rabbit anti-glyceraldehyde-3-phosphate dehydrogenase (GAPDH, 14C10, #2118) were from Cell Signaling Technology (Beverly, MA). IRDye 800CW goat anti-mouse (926-32210) and anti-rabbit IgG (926-32211) secondary antibodies were from LI-COR Biosciences (Lincoln, NE).

### Animals and treatment

Experimental protocols complied with the National Institutes of Health Guidelines on the Use of Laboratory Animals, and were approved by the Institute’s Animal Ethics Committee. Male diabetic (*db/db*) mice and their non-diabetic littermates (*db/+*) were obtained from the SLAC Experimental Animal Center (Shanghai, China) and were housed at 22 ± 1°C, adherent to a 12 hour light-dark cycle. All the animals were provided with food and water *ad libitum*. At 8 weeks of age, the *db/+* and *db/db* mice were randomized into four groups: (1) control *db/+* mice (*db/+*); (2) *db/+* mice + GW3965 (*db/+* GW); (3) *db/db* mice (*db/db*); (4) *db/db* mice + GW3965 (*db/db* GW). The mice were treated with GW3965 (20 mg/kg intraperitoneally) or vehicle daily for 12 weeks prior to sacrifice and tissue collection. GW3965 at this dose, which was chosen based upon our pilot study data and the published literature [11,13,14], GW3965 at this dose effectively invokes LXR activity without inducing observable hemodynamic changes in animal studies [11,13,14]. After 12 weeks, animals were subjected to hemodynamic measurements (described below), and hearts were excised and snap-frozen in liquid nitrogen for biochemical determinations, or fixed in formalin for histological evaluations. At the endpoint, mice were fasted overnight and serum samples were collected. Plasma glucose, total cholesterol (TC), and serum triglyceride (TG) were determined by an auto-biochemical analysis system (Chemix-180, Sysmex, Japan).

### Western blot analysis

Proteins were prepared per standard protocol, and protein lysate concentrations were determined via Pierce BCA Protein Assay Kit (Thermo Scientific, Rockford, IL). To prepare the nuclear or cytosolic fractions, protein lysate was collected via NE-PER Nuclear Protein Extraction Kit (Thermo Scientific). Equal quantities of proteins (30-50 μg/lane) were subjected to 10 or 12% SDS-PAGE, dependent upon the target proteins, electrotransferred onto nitrocellulose membranes, and incubated with primary antibodies against LXRα (1:1000), LXRβ (1:1000), cleaved caspase-3 (1:1000), NF-κB p65 (1:1000), Akt (1:1000), phospho-Akt (1:1000), p38 MAPK (1:1000), phospho-p38 MAPK (1:1000), JNK (1:1000) and phospho-JNK (1:1000). GAPDH and Histone levels were utilized as loading controls for total and nuclear protein expression, respectively. After incubation with the corresponding secondary antibodies, protein bands were detected by an Odyssey® IR scanner (LI-COR Biosciences, Lincoln, NE). Quantitation was performed via Quantity One 4.4.0 software (Bio-Rad, Hercules, CA).

### Real-time quantitative PCR

Total RNA was isolated from tissues with TRIzol Reagent and purified with Qiagen’s RNeasy Mini Kit (Qiagen, Hilden, Germany). Reverse transcription was performed by Omniscript RT Kit (Qiagen). The resultant cDNA was amplified by SYBR® Premix Ex Taq™ Perfect Real Time Kit (Takara BIO, Otsu, Japan). The PCR reaction was directly monitored by The LightCycler® 480 Real-Time PCR System (Roche Applied Science, Indianapolis, IN). Real-time PCR primers used were as follows: mouse LXRα (GenBank Accession No. NM001177730 and NM013839), forward 5′-GCTCATTGCCATCAGCATC-3′ and reverse 5′-AGCATCCGTGGGAACATCA-3′; mouse LXRβ (GenBank Accession No. NM009473 and XM_001002072), forward 5′-TGCCAGGGTTCTTGCAGTTG-3′ and reverse 5′-AACGTGATGCATTCTGTCTCGTG-3′; mouse transforming growth factor beta1 (TGF-β1) (GenBank Accession No. NM_011577), forward 5′-TACGGCAGTGGCTGAACCAA-3′ and reverse 5′-CGGTTCATGTCATGGATGGTG-3′; mouse collagen, type I, alpha 1 (collagen 1A1) (GenBank Accession No. NM_007742), forward 5′-CACCTACAGCACCCTTGTGG-3′ and reverse 5′-GGGAGGTCTTGGTGGTTTTG-3′; mouse nicotinamide adenine dinucleotide phosphate oxidase gp^91phox^ subunit (NADPH oxidase gp^91phox^ subunit) (GenBank Accession No. NM_007807), forward 5′-TGATCCTGCTGCCAGTGTGTC-3′ and reverse 5′-GTGAGGTTCCTGTCCAGTTGTCTTC-3′; mouse inducible nitric oxide synthase (iNOS) (GenBank Accession No. NM_010927), forward 5′-CAAGCTGAACTTGAGCGAGGA-3′ and reverse 5′-TTTACTCAGTGCCAGAAGCTGGA-3′; mouse GAPDH (GenBank Accession No. BC083149), forward 5′- TGGCACAGTCAAGGCTGAGA-3′ and reverse 5′-CTTCTGAGTGGCAGTGATGG-3′. Real-time PCR data were represented as Ct values, defined as the crossing threshold of PCR, obtained via LightCycler 480 Data Analysis software. The fold change in the sample gene expression was calculated after adjusting for GAPDH using the 2 ^–ΔΔCt^ method [15].

### Glucose Tolerance Test (GTT) and Homeostasis Model Assessment for Insulin Resistance (HOMA-IR)

Briefly, mice were fasted overnight (14-16 h). GTT was performed as described previously [16,17]. Glucose solution was administered via an intraperitoneal injection at a dose of 2 g/kg body weight, and the blood glucose level was measured from tail snipping at 0, 30, 60, 90, and 120 min after the initial glucose loading. Blood glucose level was determined using a One-Touch Profile portable blood glucose monitor (Roche, Mannheim, Germany). The area under the curve of the glucose concentrations (AUCg) was calculated. Serum insulin was measured by ELISA (Millipore, Billerica, MA) [18]. HOMA-IR was calculated using the following formula: HOMA-IR (mmol/L × μU/mL) = fasting glucose (mmol/L) × fasting insulin (μU/mL) /22.5 [18].

### *In situ* detection of apoptosis in heart tissue

Myocardial apoptosis was determined by terminal deoxynucleotidyl transferase dUTP nick-end labeling (TUNEL) technique via an In Situ Cell Death Detection Kit (Roche Diagnostics) as described previously [19,20]. Results were expressed as the percentage of apoptotic cells among the total cell population.

### Detection of caspase-3 activity in heart tissue

Cardiac caspase-3 activity was measured via caspase-3 Colorimetric Assay Kit (Millipore, Billerica, MA) as previously described [15,21,22]. Briefly, 100 μg of total protein from tissues was loaded and incubated with 25 μg Ac-DEVD-pNA as a colorimetric-specific substrate at 37°C for 1.5 hours. Then, pNA cleaved from DEVD by caspase-3 was quantified by a microplate reader (BioTek, Winooski, VT) at 405 nm. Changes of caspase-3 activity in *db/db* tissue samples were calculated and compared with the mean value from control *db/+* mice tissue. Data were expressed as nmol pNA /h /mg protein.

### Hemodynamic measurements

Left ventricular hemodynamics was evaluated after 12 weeks by catheterization as previously described [23]. In brief, a micromanometer-tipped catheter (1.4 F, SPR 835; Millar Instruments, Houston, TX) was inserted through the right carotid artery into the aorta of an anaesthetized mouse and carefully introduced into the left ventricle (LV). The transducer was connected to a Power Laboratory system (AD Instruments, Castle Hill, New South Wales, Australia) and variables derived from catheterization included maximal ascending and descending rates of left ventricular pressure (±dP/dt), left ventricular end-systolic pressure (LVSP), left ventricular end-diastolic pressure (LVEDP), and heart rate.

### Echocardiographic measurements

At 20 weeks of age, mice were anaesthetized with 1.5% isoflurane, and two-dimensional echocardiographic views of the mid-ventricular short axis were obtained at the level of the papillary muscle tips below the mitral valve (Vevo 770, VisualSonic, Toronto, Canada). Variables measured on M-mode echocardiography including LV wall thickness, LV chamber dimensions, and systolic and diastolic function were analyzed as described previously [24].

### Sirius red staining

LV myocardial sections were stained with 0.1% picric sirius red (Sigma-Aldrich, St Louis, MO) for fibrosis detection, as previously described [25]. The severity of cardiac fibrosis was evaluated after sirius red staining at 20 × magnification with the use of Image-pro plus 6.1 software (Media Cybernetics, Bethesda, MD). Collagen-positive area was normalized to the total cross-sectional area of left ventricle and was expressed as a percentage. Areas containing blood vessels and perivascular interstitial cells were excluded from fibrosis quantification [26].

### Measurement of oxidative stress generation in heart tissue

Myocardial reactive oxygen species (ROS) generation was measured by confocal microscope via *in situ* DHE stain or lucigenin-enhanced chemiluminescence. For DHE stain, unfixed frozen cross-sections (5 μm) was incubated with DHE (5 μmol/L) at 37°C for 30 minutes in a humidified chamber protected from light, followed by 5 minutes of PBS washing to remove non-intercalated ethidium bromide molecules. Images were obtained and analyzed via Leica laser scanning confocal microscope (Leica TCS SP5 II). NADPH oxidase activity within the heart homogenates was measured by lucigenin-enhanced chemiluminescence via luminometer as previously described [11,21,22]. The lucigenin concentration in the final reaction mixture was 0.25 mmol/L, and NADPH-dependent superoxide production was expressed as relative light units (RLU) per mg per second (RLU • mg^-1^ • s^-1^).

### Determination of nitrative stress generation in heart tissue

Myocardial reactive nitrative stress (RNS) was assessed by nitrotyrosine content, a footprint of *in vivo* peroxynitrite formation [11,21,22], by both immunostaining and enzyme-linked immunosorbent assay (ELISA). For immunostaining, paraffin-embedded slices were stained with primary antibody against nitrotyrosine (1:100), and then immunostained by Vectastain ABC kit (Vector Laboratories, Burlingame, CA; 1:200). For ELISA, cardiac tissue nitrotyrosine content was quantified by Nitrotyrosine ELISA Kit (Abnova, Taiwan). Results were expressed as nanomoles/g protein.

### Assessment of inflammatory cytokines in cardiac tissue

Tumor necrosis factor alpha (TNF-α) and interleukin 1-beta (IL-1β) were quantified using an ELISA kit (Invitrogen, Camarillo, CA) per manufacturer’s instructions. The tissue supernatant fluids were added to each well, and treated with detection antibody, supplemented with substrate and stop solution. TNF-α and IL-1β levels were determined by microplate reader (450 nm).

### Statistical analysis

All values in the text and figures are presented as the mean ± SEM of independent experiments from given n-sizes. Statistical significance of multiple treatments was determined by one-way analysis of variance (ANOVA) followed by Tukey’s *post hoc* analysis using GraphPad Prism 5 software (San Diego, California). Probabilities of 0.05 or less were considered to be statistically significant (2-tailed).

## Results

### Both LXRα and LXRβ are expressed in adult heart tissue, but LXRα is selectively upregulated by hyperglycemia in db/db mice

**Figure 1:**
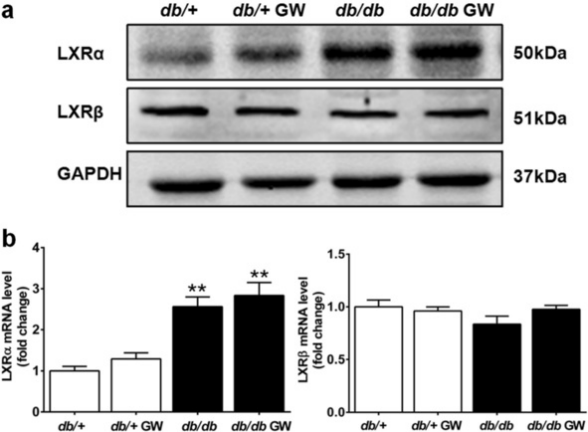
**LXRs expression in cardiac tissue. a**-**b**. LXRα and LXRβ expression in cardiac tissue from *db/+*, *db/+* GW, *db/db* and *db/db* GW mice were determined by Western blot and real-time PCR (n =5-6). **P < 0.01 *vs db/+* group. Abbreviations: GW, GW3965.

Both LXRα and LXRβ subtypes were detected in cardiac tissue, as demonstrated by both Western blot (Figure 1a) and real-time PCR (Figure 1b). Interestingly, endogenous LXRα protein level significantly increased in the *db/db* and *db/db* GW group, whereas LXRβ expression remained mostly unaffected.

### LXR agonist GW3965 attenuates the phenotype of type 2 diabetes in db/db mice

We first investigated whether the activation of LXR could suppress hyperglycemia and other metabolic abnormalities in diabetic mice. As illustrated in Table 1, the *db/db* group weighed more than the *db/+* group, and demonstrated derangements of serum metabolic markers such as glucose, TC and TG. Chronic treatment of 8-week-old male diabetic *db/db* mice with LXR agonist GW3965 lowered body weight, blood glucose levels, and plasma TC levels. However, GW3965 showed no influence on the increased plasma TG level in diabetic mice. These findings indicate that LXR activation by GW3965 improved the metabolic disorders in this mouse model of T2DM.

**Table 1:** **GW3965 attenuated diabetes-induced metabolism abnormalities**

**Group**	**Body weight**	**Blood glucose**	**TC**	**TG**
	**(g)**	**(mmol/L)**	**(mmol/L)**	**(mmol/L)**
***db/+***	27.84 ± 0.52	7.00 ± 0.53	2.08 ± 0.04	0.89 ± 0.02
***db/+*** **GW**	27.76 ± 0.60	5.30 ± 0.56	2.13 ± 0.13	0.97 ± 0.02
***db/db***	54.85 ± 0.70*	19.93 ± 1.56*	4.31 ± 0.15*	1.56 ± 0.04*
***db/db*** **GW**	45.28 ± 0.66*^#^	7.00 ± 0.97^**#**^	2.91 ± 0.13*^#^	1.71 ± 0.04*

Fasting blood glucose, TC, TG levels and body weight were all measured on the day of animal sacrifice. Data are means **±** SEM; *P < 0.05 *vs db/+* group; ^**#**^P < 0.05 *vs db/db* group; n =8-10. *Abbreviations*: *GW* GW3965, *TC* total cholesterol, *TG* triglycerides.

### LXR agonist GW3965 improves glucose tolerance and insulin sensitivity in db/db mice

**Figure 2:**
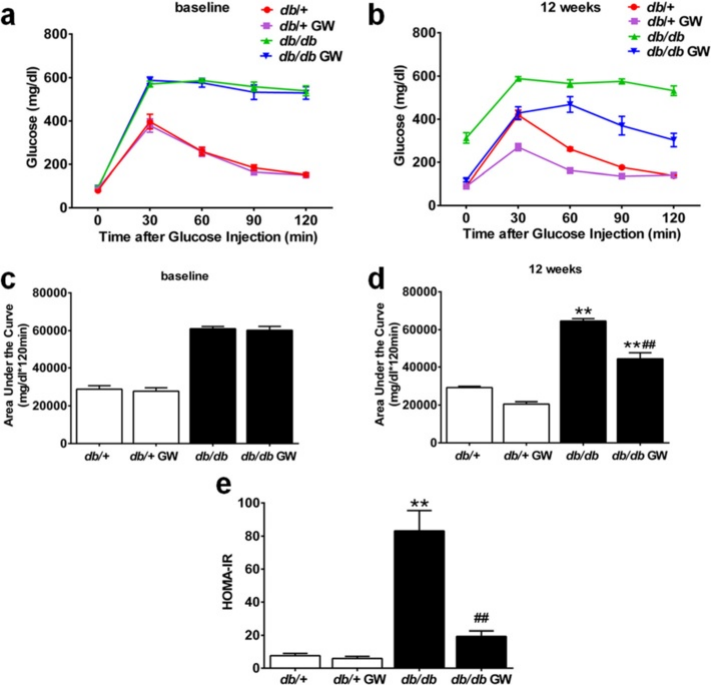
**GW3965 improved glucose tolerance and insulin sensitivity in** ***db/db*** **mice. a**-**b**. GTT was performed at baseline and 12 weeks after treatment with GW3965 (n =7-9). **c**-**d**. Area under the curve of glucose levels during the GTT were also calculated (n =7-9 per group). **e**. HOMA-IR index was detected (n =7-9). **P < 0.01 *vs db/+* group; ^##^ *P* < 0.01 *vs db/db* group. Abbreviations: GTT, glucose tolerance test; GW, GW3965; HOMA-IR, homeostasis model assessment for insulin resistance.

We next tested the effect of LXR agonist on insulin resistance in *db/db* diabetic mice. At 20 weeks of age, there were no statistically significant differences in the GTT, the AUCg and HOMA-IR index between the *db/+* and *db/+* GW group (Figure 2). However, the levels of GTT, AUCg, and HOMA-IR index were markedly deteriorated in *db/db* diabetic mice, which were significantly reversed by the administration of GW3965 for 12 weeks (Figure 2).

### LXR agonist GW3965 inhibits myocardial apoptosis and cardiac dysfunction in db/db mice

**Figure 3:**
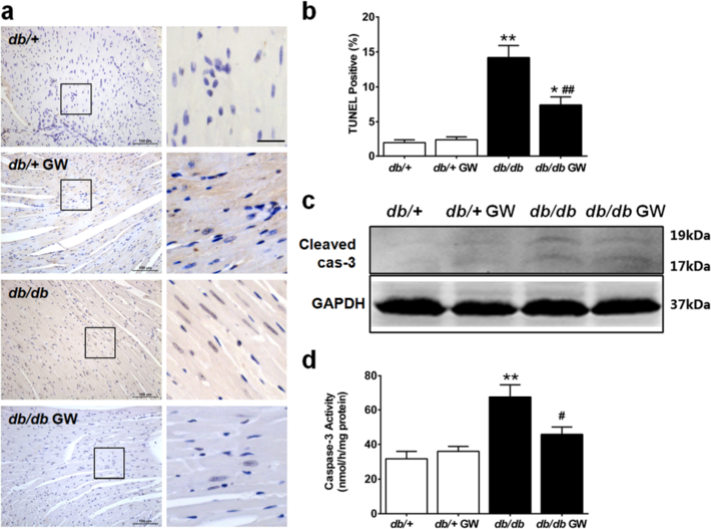
**GW3965 inhibited diabetes-induced myocardial apoptosis. a**. Representative image of TUNEL immunostaining. Left panel: 20 × Magnification, scale bar 100 μm. Right panel: TUNEL stain of box of left panel; 40 × Magnification, scale bar 25 μm. TUNEL positive cells were brown. **b**. The percentage of TUNEL positive cells was calculated. **c**-**d**. Myocardial apoptosis was determined by Western blot analysis of cleaved caspase-3 (n =5-6) and quantification of caspase-3 activation (n =6-10). *P < 0.05 or **P < 0.01 *vs db/+* group; ^#^ *P* < 0.05 or ^##^ *P* < 0.01 *vs db/db* group. Abbreviations: GW, GW3965; TUNEL, terminal deoxynucleotidyl transferase dUTP nick end labeling; cas-3, caspase-3.

**Figure 4:**
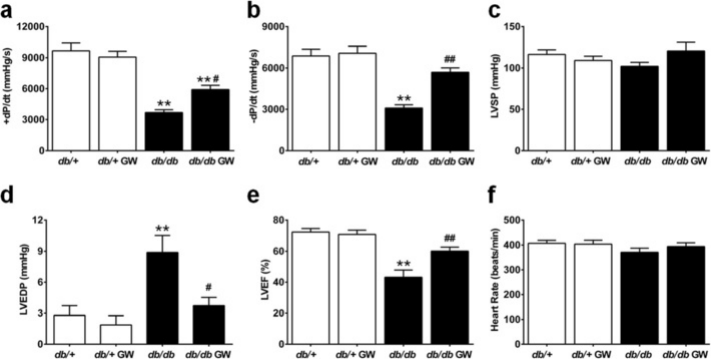
**GW3965 limited diabetes-induced left ventricular dysfunction. a**-**f**. Left ventricular function was assessed via echocardiography and cardiac catheterization in anesthetized mice (n =6-8). **P < 0.01 *vs db/+* group; ^#^ *P* < 0.05 or ^##^ *P* < 0.01 *vs db/db* group. Abbreviations: GW, GW3965; ±dP/dt, maximal ascending and descending rates of LV pressure; LVSP, left ventricular systolic pressure; LVEDP, left ventricular end-diastolic pressure; LVEF, left ventricular ejection fraction.

In an attempt to determine the cardioprotective role of LXR agonist, we investigated the effects of GW3965 on cellular apoptosis and cardiac function in type 2 diabetic *db/db* mice. Our results demonstrated that compared with the *db/+* group, *db/db* mice clearly showed more TUNEL-positive particles (Figure 3, a and b), increased cleaved caspase-3 expression, enhanced caspase-3 activity (Figure 3, c and d) in cardiomyocytes, and deteriorated left ventricular dysfunction (Figure 4). GW3965 treatment significantly decreased cardiomyocyte apoptosis and improved cardiac function (Figures 3 and 4).

### LXR agonist GW3965 reduces cardiac fibrosis in db/db mice

**Figure 5:**
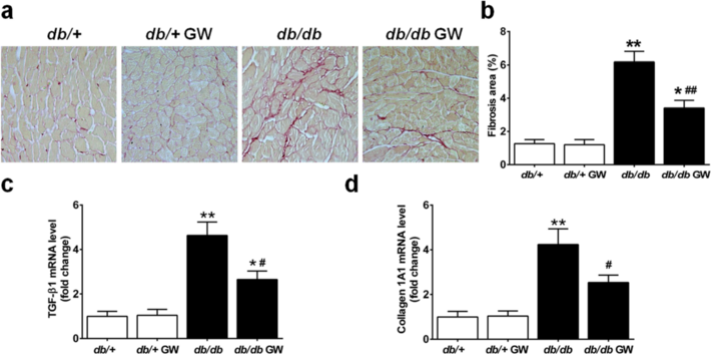
**GW3965 reduced diabetes-induced cardiac fibrosis. a**. Representative image of Sirius Red-stained LV sections, 20 × Magnification. **b**. Pooled data on quantification of collagen area per visual field (n =8-9). **c**-**d**. TGF-β1 and collagen 1A1 gene expression was determined by real-time PCR. Results were normalized against GAPDH and converted to fold induction relative to *db*/+ mice (n =6). *P < 0.05 or **P < 0.01 *vs db/+* group; ^#^ *P* < 0.05 or ^##^ *P* < 0.01 *vs db/db* group. Abbreviations: GW, GW3965; LV, left ventricle; TGF-β1, transforming growth factor beta1; collagen 1A1, collagen, type I, alpha 1.

By sirius red staining, we found that *db/db* mice exhibited more severe cardiac fibrosis than the control group (Figure 5, a and b). This induction of fibrosis was significantly ameliorated after GW3965 treatment for 12 weeks (Figure 5, a and b). Moreover, real-time PCR analysis revealed significant increases in the expression of profibrotic genes (TGF-β1 and collagen-1A1) in diabetic hearts, which were attenuated by GW3965 (Figure 5, c and d).

### LXR agonist GW3965 attenuates myocardial oxidative stress and nitrative stress in db/db mice

**Figure 6:**
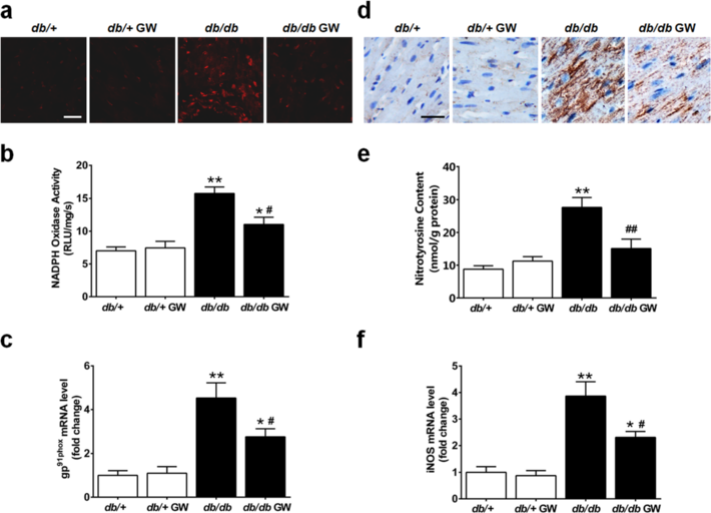
**GW3965 attenuated diabetes-induced myocardial oxidative/nitrative stress. a**-**c**. GW3965 attenuated oxidative stress in the myocardial tissues. **a**. Myocardial oxidative stress was measured by confocal microscopy with *in situ* dihydroethidium stain (n =5-6, scale bar 25 μm). **b**. NADPH oxidase activity was determined by lucigenin-enhanced chemiluminescence (n =6-11). **c**. NADPH oxidase gp^91phox^ gene expression was determined by real-time PCR (n =6). Results were normalized against GAPDH and converted to fold induction relative to *db*/+ mice. **d**-**f**. GW3965 attenuated nitrative stress in the myocardial tissues. **d**. Myocardial nitrative stress was assessed via nitrotyrosine levels determined by immunohistochemistry (n =5-6, scale bar 25 μm). **e**. Myocardial nitrotyrosine content was determined by ELISA analysis (n =6-11). **f**. iNOS gene expression was determined by real-time PCR (n =6). Results were normalized against GAPDH and converted to fold induction relative to *db*/+ mice. *P < 0.05 or **P < 0.01 *vs db/+* group; ^#^ *P* < 0.05 or ^##^ *P* < 0.01 *vs db/db* group. Abbreviations: GW, GW3965; RLU, relative light units; iNOS, inducible nitric oxide synthase.

To further determine the underlying mechanisms of LXR agonist’s protective action, we investigated the effects of GW3965 on oxidative/nitrative stress in the diabetic myocardium. GW3965 significantly attenuated ROS production in *db/db* mice (Figure 6, a and b) and inhibited the expression of the NADPH oxidase subunit gp^91phox^ (Figure 6c). Moreover, GW3965 significantly reduced tissue nitrotyrosine content (a well-accepted footprint of *in vivo* nitrative stress, Figure 6, d and e), and inhibited iNOS expression (Figure 6f). Collectively, these results demonstrate that GW3965 attenuated diabetes-induced oxidative/nitrative stress.

### LXR agonist GW3965 suppresses diabetes-induced myocardial nuclear factor-κB activation and inflammation

**Figure 7:**
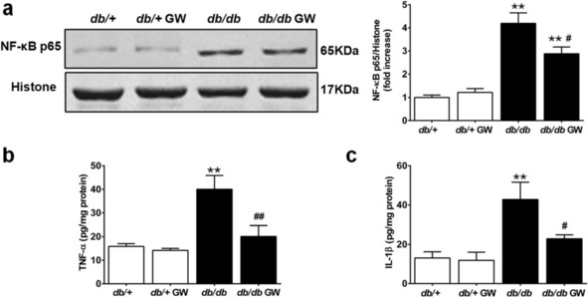
**GW3965 suppressed diabetes-induced inflammatory response in the myocardial tissues. a**. Expression of nuclear NF-κB p65 subunit in the myocardial tissue was determined by Western blot (n =5-6). Histone level served as loading control for nuclear protein expression. Results were normalized against histone and converted to fold induction relative to *db*/+ mice. **b**-**c**. TNF-α and IL-1β content in the myocardial tissue was determined by ELISA analysis (n =6-8). **P < 0.01 *vs db/+* group; ^#^ *P* < 0.05 or ^##^ *P* < 0.01 *vs db/db* group. Abbreviations: GW, GW3965; NF-κB, nuclear factor kappa-light-chain-enhancer of activated B; TNF-α, tumor necrosis factor-α; IL-1β, interleukin-1β.

To investigate whether LXR agonist provided cardioprotection by inhibiting the inflammatory response in the diabetic myocardium, we investigated the effect of GW3965 on inflammatory cytokine production. Protein levels of NF-κB p65 were markedly increased in the myocardium of *db/db* mice (Figure 7a). The expression of proinflammatory cytokines, including TNF-α and IL-1β, was significantly augmented in *db/db* mice (Figure 7, b and c). Furthermore, treatment with GW3965 significantly decreased nuclear NF-κB p65 expression and proinflammatory cytokine (TNF-α and IL-1β) levels in *db/db* mice (Figure 7). These results indicate that the anti-inflammatory effects of LXR agonist contribute to cardioprotection against DCM.

### LXR agonist GW3965 ameliorates the impairment of insulin/Akt signaling pathway and mitigates diabetes-induced activation of MAP kinases

**Figure 8:**
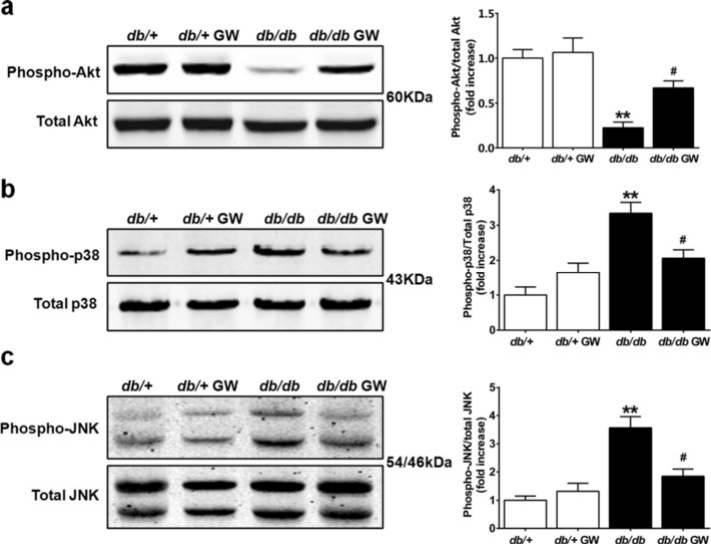
**GW3965 differentially regulated Akt and MAP kinases activation. a**-**c**. Western blot analysis shows the phosphorylation and protein expression of Akt, p38 MAPK and JNK expression in the myocardial tissues (n =5-6). **P < 0.01 *vs db/+* group; ^#^ *P* < 0.05 *vs db/db* group. Abbreviations: GW, GW3965; p38 MAPK, p38 mitogen-activated protein kinase; JNK, c-Jun N- terminal kinase.

To further investigate the cellular mechanisms by which LXR activation may attenuate diabetes-induced insulin resistance and cardiac oxidative stress, we evaluated insulin/Akt signaling and MAPK pathways, which are the most important pathways involved in insulin resistance and oxidative/nitrative stress. Serine phosphorylation of Akt (serine 473) was impaired in the myocardium of *db/db* mice. However, 12-week treatment with GW3965 significantly restored Akt activation in *db/db* mice (Figure 8a). Likewise in diabetic myocardium, an increase in the activation of p38 MAPK (Figure 8b) and JNK (Figure 8c) could be observed. Treatment with GW3965 significantly inhibited diabetes-induced p38 MAPK and JNK phosphorylation without altering their protein levels (Figure 8, b and c). These data suggest that LXR activation alleviated DCM mainly by modulating Akt and MAP kinases pathways in type 2 diabetes.

## Discussion

The major findings emanating from the current study are as follows: (1) Both LXRα and LXRβ subtypes were detected in adult cardiac tissue, but LXRα was selectively upregulated by hyperglycemia; (2) GW3965 ameliorated metabolism and improved glucose tolerance and insulin sensitivity; (3) GW3965 protected the heart in the *db/db* mouse model of type 2 diabetes from the development of diastolic dysfunction, cell death, and cardiac fibrosis; and (4) GW3965 also restored Akt phosphorylation and inhibited MAP kinases phosphorylation, and reduced oxidative/nitrative stress and inflammation response in the diabetic myocardium. Taken together, these results suggest that LXR agonist GW3965 may have great therapeutic potential in the treatment of DCM.

Originally cloned from a rat liver cDNA library and identified as an orphan nuclear hormone receptor [27], LXR is highly expressed in the enterohepatic tissue, playing a pivotal role in cholesterol and lipid metabolism, glucose homeostasis, and inflammatory response [5]. Recent evidence suggests the presence of LXR in the cardiovascular system and its significant role in cardiovascular physiology/pathology [8-11]. In the myocardium, the expression of LXRα was increased in streptozotocin-induced diabetic rats [28]. LXR agonist T0901317 attenuated high glucose-induced cardiomyocyte apoptosis *in vitro* [12]*.* However, the role of LXR in DCM *in vivo* remains unknown. To the best of our knowledge, the current study provides the first direct evidence demonstrating that GW3965 protects cardiomyocytes against hyperglycemia-induced chronic adverse changes *in vivo*. These data support the notion that LXR agonist may serve as a novel therapeutic modality in the management of DCM.

Hyperglycemia and insulin resistance have long been considered a central component in the pathogenesis of DCM [29,30]. In insulin resistance states, impaired myocardial glucose uptake has been shown to provoke myocardial dysfunction [31]. In addition, insulin resistance independently predicts mortality in diabetic patients with heart failure [3]. Multiple lines of evidence have indicated that LXR is an important regulator of glucose metabolism in different animal models of T2DM. Previous studies showed that pharmacologic LXR activation by GW3965 improved glucose tolerance by limiting hepatic glucose output and improving peripheral glucose uptake in a murine model of diet-induced obesity and insulin resistance [32]. Moreover, LXR agonist GW3965 reduced blood glucose concentrations and improved insulin sensitivity in *ob/ob* mice [33]. The results of the present study further demonstrated that the LXR agonist GW3965 ameliorates systemic insulin resistance and myocardial dysfunction in a *db/db* murine model.

Hyperglycemia and insulin resistance contribute to the generation of excessive ROS/RNS which have damaging effects on myocardial function. Hyperglycemia enhances glucose oxidation and mitochondrial generation of ROS, which has been implicated as a key stimulator of these cardiac impairments [24,34,35]. Meanwhile, NO is overproduced by activated iNOS, reacts with ROS, and turns into the highly reactive ONOO^-^ (RNS). ROS/RNS trigger myocardial apoptosis, and damage the mitochondrial membrane, leading to a “ROS-induced ROS release” vicious cycle, which further worsens myocardial damage and dysfunction [3,36]. Because of these roles of ROS/RNS, reducing oxidative/nitrative stress should be favored in the management of DCM. In this report, we demonstrate that long-term administration of GW3965 significantly decreased the expression of myocardial NADPH oxidase, as well as superoxide production and tissue nitrotyrosine content (the footprint of *in vivo* nitrative stress). Furthermore, we showed that GW3965 treatment inhibited the downstream inflammatory response (i.e., activation of nuclear NF-κB and pro-inflammatory cytokines) in diabetic myocardium *in vivo*. Thus, inhibiting ROS/RNS and inflammatory pathways could be an important mechanism responsible for LXR agonist-mediated cardioprotection against DCM.

There is accumulating evidence that in the setting of type 2 diabetes, insulin resistance and ROS/RNS may be coconspirators in cardiac dysfunction, each capable of triggering or worsening the other [37]. The insulin-Akt signaling and MAPK are the most important pathways involved in insulin resistance and oxidative stress [38]. It has been demonstrated the synthetic LXR agonists ameliorated insulin resistance by restoring the insulin-Akt signalling cascade and preventing JNK activation in adipocytes [39,40]. In the current study, the insulin-Akt pathway was blunted, compared with the activated MAPK pathway in the myocardium of *db/db* mice. Treatment with GW3965 restored Akt activation and inhibited MAP kinases phosphorylation in the diabetic myocardium, suggesting that differential regulation of Akt and MAP kinases activation are likely responsible for the aforementioned cardioprotective effects of the LXR agonist against DCM; further studies are warranted to define in more detail the complex mechanisms involved in regulation of oxidative stress, inflammatory response, and the cardioprotective effect of GW3965.

## Conclusion

**Figure 9:**
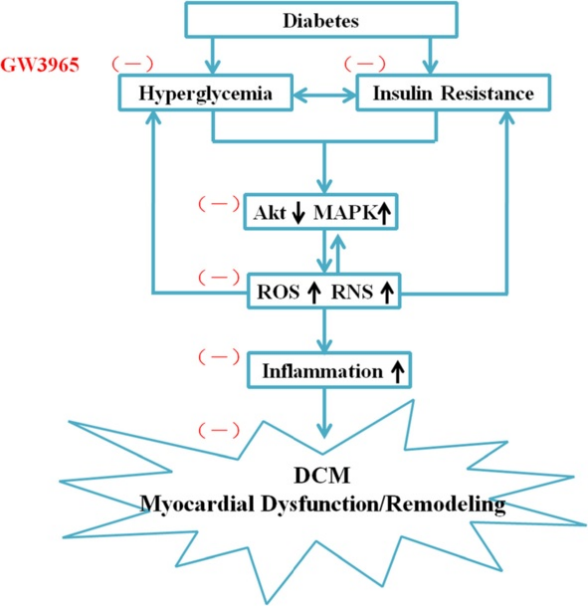
**Proposed scheme for the cardioprotective effect of LXR agonist GW3965 against DCM.** Abbreviations: MAPK, mitogen-activated protein kinase; ROS, reactive oxygen species; RNS, reactive nitrative stress; DCM, diabetic cardiomyopathy.

Our data demonstrate that the LXR agonist GW3965 exerts a protective effect on DCM by (at least in part) attenuating insulin resistance, modulating Akt and MAP kinases pathways, and reducing oxidative/nitrative stress and inflammatory response (Figure 9). LXR, therefore, is a potentially attractive molecular target for the treatment of DCM.

## Abbreviations

AUCg: Area under the curve of the glucose concentrations
DCM: Diabetic cardiomyopathy
DHE: Dihydroethidium
±dP/dt: Ascending and descending rates of left ventricular pressure
ELISA: Enzyme-linked immunosorbent assay
GLUT4: Glucose transporter 4
HOMA-IR: Homeostasis model assessment- insulin resistance
GAPDH: Glyceraldehyde-3-phosphate dehydrogenase
GTT: Glucose tolerance test
IL-1β: Interleukin 1-beta
iNOS: Inducible nitric oxide synthase
JNK: c-Jun N-terminal kinase
LV: Left ventricle
LVEF: Left ventricular ejection fraction
LVEDP: Left ventricular end-diastolic pressure
LVSP: Left ventricular end-systolic pressure
LXR: Liver X receptor
MAP: Kinase (MAPK) mitogen-activated protein kinase
NADPH: Oxidase nicotinamide adenine dinucleotide phosphate oxidase
NF-κB: Nuclear factor kappa-light-chain-enhancer of activated B
p38 MAPK: p38 mitogen-activated protein kinase
RLU: Relative light units
RNS: Reactive nitrogen species
ROS: Reactive oxygen species
T2DM: Type 2 diabetes mellitus
TC: Total cholesterol
TG: Triglyceride
TGF-β1: Transforming growth factor beta1
TNF-α: Tumor necrosis factor alpha
TUNEL: Terminal transferase dUTP nick end labeling
